# Physiology-guided PCI versus CABG for left main coronary artery disease: insights from the DEFINE-LM registry

**DOI:** 10.1007/s12928-023-00932-z

**Published:** 2023-04-05

**Authors:** Takayuki Warisawa, Christopher M. Cook, Yoshiaki Kawase, James P. Howard, Yousif Ahmad, Henry Seligman, Christopher Rajkumar, Takumi Toya, Shunichi Doi, Akihiro Nakajima, Toru Tanigaki, Hiroyuki Omori, Masafumi Nakayama, Rafael Vera-Urquiza, Sonoka Yuasa, Takao Sato, Yuetsu Kikuta, Hidetaka Nishina, Rasha Al-Lamee, Sayan Sen, Amir Lerman, Yoshihiro J. Akashi, Javier Escaned, Hitoshi Matsuo, Justin E. Davies

**Affiliations:** 1 Division of Cardiology, Department of Internal Medicine, St. Marianna University School of Medicine, 2-16-1 Sugao, Kawasaki City, Kanagawa Prefecture 216-8511 Japan; 2 Department of Cardiovascular Medicine, NTT Medical Center Tokyo, Tokyo, Japan; 3 National Heart and Lung Institute, Imperial College London, London, UK; 4 The Essex Cardiothoracic Centre, Basildon, UK; 5 Anglia Ruskin University, Chelmsford, UK; 6 Department of Cardiovascular Medicine, Gifu Heart Center, Gifu, Japan; 7 Cardiovascular Science, Hammersmith Hospital, Imperial College Healthcare NHS Trust, London, UK; 8 Cardiovascular Medicine, Yale School of Medicine, New Haven, USA; 9 Department of Cardiology, National Defense Medical College, Tokorozawa, Japan; 10 Department of Cardiovascular Medicine, Mayo Clinic, Rochester, USA; 11 Department of Cardiovascular Medicine, New Tokyo Hospital, Matsudo, Japan; 12 Cardiovascular Center, Toda Central General Hospital, Toda, Japan; 13 Cardiovascular Institute, Hospital Clínico San Carlos, Madrid, Spain; 14 Department of Cardiology, Tachikawa General Hospital, Nagaoka, Japan; 15 Division of Cardiology, Fukuyama Cardiovascular Hospital, Fukuyama, Japan; 16 Department of Cardiology, Tsukuba Medical Center Hospital, Tsukuba, Japan

**Keywords:** Coronary physiology, Instantaneous wave-free ratio, Left main coronary artery disease, Registry-based study

## Abstract

There have been no studies comparing clinical outcomes of physiology-guided revascularization in patients with unprotected left main coronary disease (ULMD) between percutaneous coronary intervention (PCI) vs. coronary artery bypass grafting (CABG). The aim of this study was to assess the long-term clinical outcomes between PCI and CABG of patients with physiologically significant ULMD. From an international multicenter registry of ULMD patients interrogated with instantaneous wave-free ratio (iFR), we analyzed data from 151 patients (85 PCI vs. 66 CABG) who underwent revascularization according to the cutoff value of iFR ≤ 0.89. Propensity score matching was employed to adjust for baseline clinical characteristics. The primary endpoint was a composite of all-cause death, non-fatal myocardial infarction, and ischemia-driven target lesion revascularization. The secondary endpoints were the individual components of the primary endpoint. Mean age was 66.6 (± 9.2) years, 79.2% male. Mean SYNTAX score was 22.6 (± 8.4) and median iFR was 0.83 (IQR 0.74–0.87). After performing propensity score matching analysis, 48 patients treated with CABG were matched to those who underwent PCI. At a median follow-up period of 2.8 years, the primary endpoint occurred in 8.3% in PCI group and 20.8% in CABG group, respectively (HR 3.80; 95% CI 1.04–13.9; *p* = 0.043). There was no difference in each component of the primary event (*p* > 0.05 for all). Within the present study, iFR-guided PCI was associated with lower cardiovascular events rate in patients with ULMD and intermediate SYNTAX score, as compared to CABG.

**Graphical abstract:**

State-of-the-art PCI vs. CABG for ULMD. Study design and primary endpoint in patients with physiologically significant ULMD. MACE was defined as the composite of all-cause death, non-fatal myocardial infarction, and target lesion revascularization. The blue line denotes the PCI arm, and the red line denotes the CABG arm. PCI was associated with significantly lower risk of MACE than CABG. CABG: coronary artery bypass grafting; iFR: instantaneous wave-free ratio; MACE: major adverse cardiovascular events; PCI: percutaneous coronary intervention; ULMD: unprotected left main coronary artery disease.

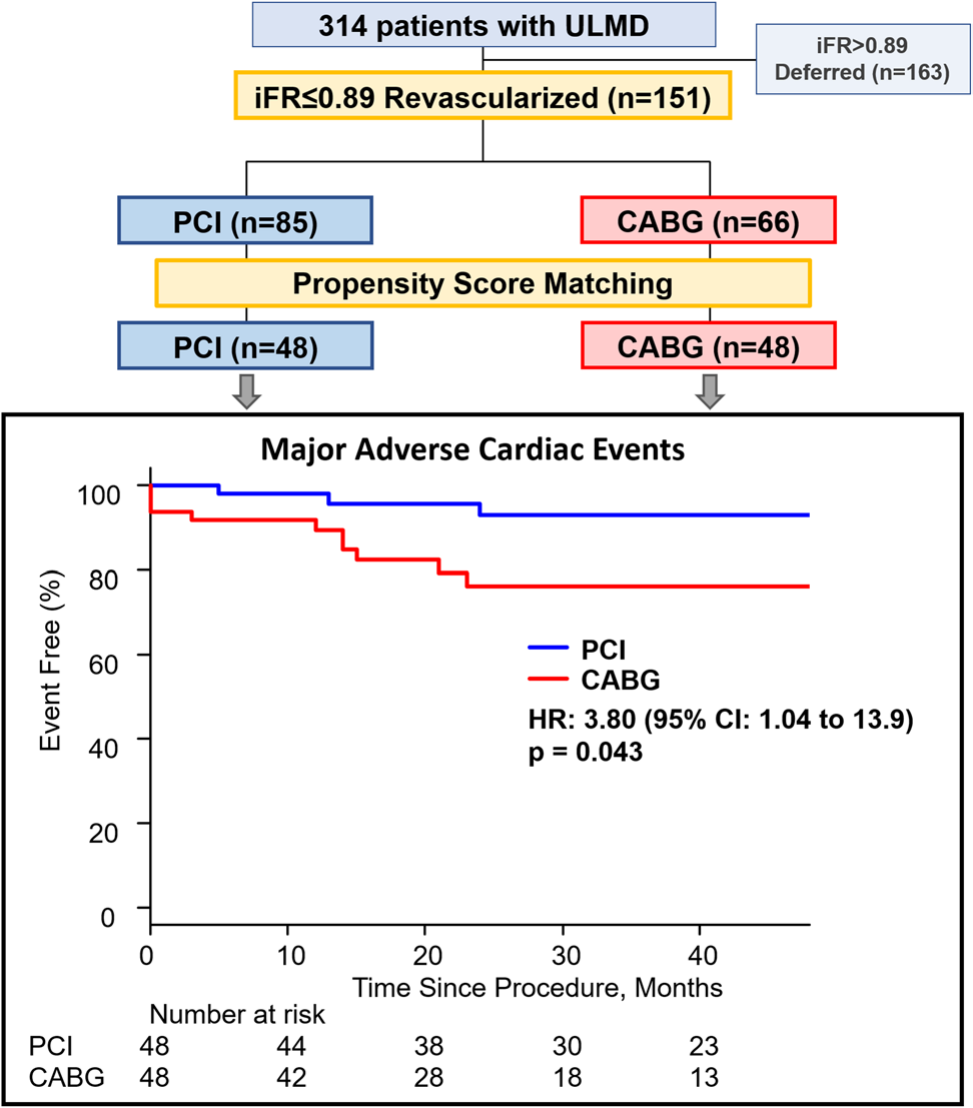

**Supplementary Information:**

The online version contains supplementary material available at 10.1007/s12928-023-00932-z.

## Introduction

Coronary physiology has a key role in revascularization decision-making in patients with stable coronary artery disease [1, 2]. Reflecting contemporary percutaneous coronary intervention (PCI) practice, including physiology-guided target lesion selection and intracoronary imaging-guided stent optimization in the era of new generation drug-eluting stent (DES) platforms, the SYNTAX-II study demonstrated significantly improved clinical outcomes in patients with three-vessel diseases compared to the predefined SYNTAX-I PCI cohort, and comparable outcomes with the cohort of coronary artery bypass grafting (CABG) cohort in the SYNTAX-I trial [3, 4]. However, since patients with unprotected left main coronary artery disease (ULMD) have largely been excluded from the majority of randomized controlled trials of physiology-guided revascularization [5–9], the impact of such a contemporary PCI strategy has not been investigated for ULMD revascularization.

In the EXCEL trial, long-term clinical outcomes between PCI and CABG revascularization methods for ULMD of low or intermediate SYNTAX score were comparable [10, 11]. However, the utilization of coronary physiology to guide revascularization in EXCEL was low (9.0%). Considering the demonstrated benefits of physiology-guided PCI in patients with stable coronary artery disease, in this study, we hypothesized that PCI may be associated with superior clinical outcomes for ULMD of intermediate anatomical complexity if revascularization was exclusively guided by coronary physiology. Accordingly, the aim of this study was to investigate the comparative long-term clinical outcomes of revascularization by PCI versus CABG in patients with hemodynamically significant ULMD determined by instantaneous wave-free ratio (iFR).

## Methods

### Study population

**Fig. 1:**
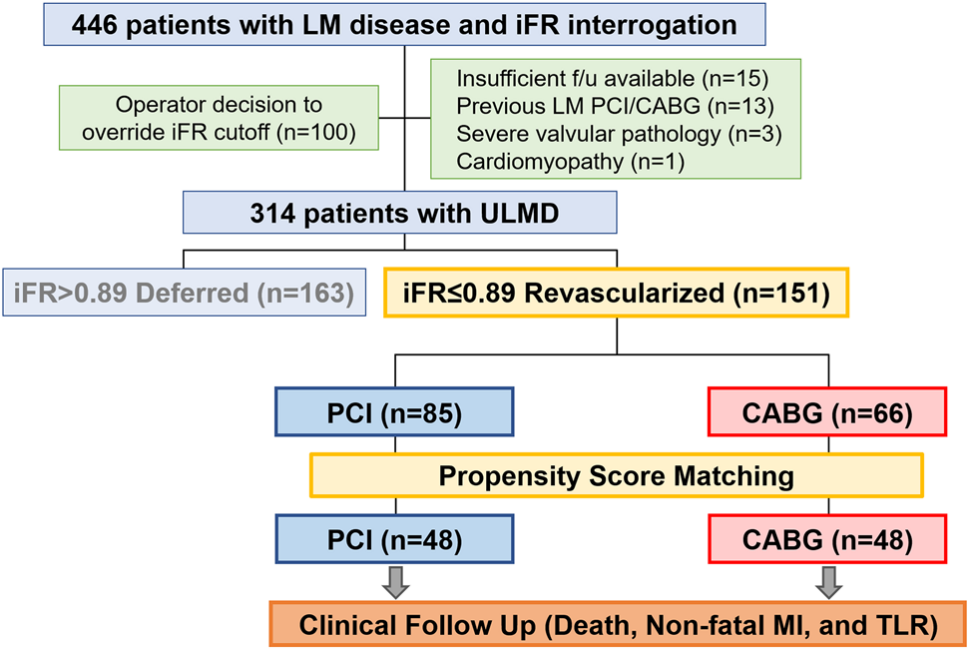
Study flow. Consecutive cases with de-novo stable ULMD of intermediate angiographic severity and physiologically significance were analyzed. Clinical outcomes were compared between PCI and CABG after propensity score matching for baseline clinical characteristics. CABG coronary artery bypass grafting, iFR instantaneous wave-free ratio, MI myocardial infarction, PCI percutaneous coronary intervention, TLR target lesion revascularization, ULMD unprotected left main coronary artery disease

As described in our previous report [12], the DEFINE-LM (deferral of coronary revascularization based on instantaneous wave-free ratio evaluation for left main coronary artery disease) registry is an international multicenter registry. Consecutive patients were included between October 2012 and October 2018 at 10 cardiac centers in Europe, the USA and Japan. Inclusion criteria were as follows: patients with stable angina; ULMD of 40–70% on visual angiographic assessment; and iFR interrogation for ULMD. Exclusion criteria were as follows: previous CABG or previous PCI for ULMD; severe valvular pathology; and any type of non-ischemic cardiomyopathy. The selection of revascularization option, i.e. PCI or CABG was decided by heart-team discussion at each participating center and revascularization decisions for non-LM disease were at the operators discretions (using physiological values and pressure-wire pullback assessment in some cases). Namely, in the present study, consecutive cases with stable ULMD of intermediate angiographic severity and physiological significance (iFR ≤ 0.89) were analyzed. The study flow diagram is shown in Fig. 1. All patients provided written informed consent. This study was approved by the local ethical committees at each participating center and was conducted according to the principles of the Declaration of Helsinki.

### Measurement of iFR

The detail of iFR measurement has been described elsewhere [12]. Specifically, iFR was measured at the distal point of LM segment either in the left anterior descending artery (LAD) or left circumflex artery (LCx). If the bifurcation lesion involved an ostial LAD or LCx, it was also considered as LM segment. If iFR was measured in both the LAD and LCx in the case of bifurcation lesion, the lower iFR value was used. When further downstream disease was present in the LAD or LCx, the wire was placed either in the non-diseased artery or proximal to the first angiographical stenosis.

### Study endpoints

The primary endpoint was the rate of major adverse cardiovascular events (MACE) over follow-up. MACE was defined as a composite of all-cause death, non-fatal myocardial infarction (MI), and ischemia-driven target lesion revascularization (TLR). Secondary endpoints were the individual components of the primary endpoint. MI included spontaneous ST-segment elevation MI and non-ST-segment elevation MI, as well as periprocedural MI. Periprocedural MI was defined by an elevation of cardiac troponin values > 5 times for PCI and > 10 times for CABG of the 99th percentile upper reference limit according to the fourth universal definition of myocardial infarction [13]. TLR was recorded as MACE when it was not the index procedure and was not identified at the time of the index procedure as a staged procedure to occur within 60 days. Patients were followed up for clinical visits at each participating center. When needed, patients or their general practitioners/family doctors were contacted for additional confirmatory clinical information.

### Statistical analysis

Categorical data are expressed as numbers and percentages. Continuous variables are expressed as mean and (±) standard deviation or as median accompanied by interquartile range (IQR) as appropriate. Continuous variables were compared with Student’s *t* or Mann–Whitney *U* tests, and categorical variables with Chi-square or Fisher exact tests, as appropriate. Propensity score matching was performed for baseline clinical characteristics using a 1:1 matching protocol, with a caliper width of 0.20 standard deviation of the propensity score (the nearest neighbor matching). Baseline clinical characteristics included age, sex, and the presence of hypertension, dyslipidemia, diabetes mellitus, chronic kidney disease, current smoking, family history of coronary artery disease, and previous MI. The dependent variable in the analysis was time to initial events during follow-up. Kaplan–Meier curves for MACE-free survival were constructed and compared between the two groups through the log-rank test, while relative differences were summarized by hazard ratios (HRs) and 95% confidence intervals (CIs) from Cox regression models. Where one arm showed no events, log-rank *p* values for those outcomes were provided alone, without HRs and associated CIs. Variables which could potentially predict MACE were analyzed by univariate and multivariate Cox regression analyses. All probability values were two-sided, and *p* values < 0.05 were considered statistically significant. All the statistical analysis was performed using R version 3.2.1 (R Foundation for Statistical Computing, Vienna, Austria).

## Results

### Study population

Among the included patients, 151 patients underwent revascularization strictly according to an iFR cutoff value of ≤ 0.89 (Fig. 1). Mean age was 67.1 ± 10.2 years (82.8% male). Mean SYNTAX score was 22.6 ± 8.4 and mean percent diameter stenosis was 49.2 ± 13.5%. The median iFR value was 0.82 (IQR 0.70–0.86). According to the heart-team discussion, revascularization was recommended and subsequently performed by either PCI (*n* = 85, 56.3%) or CABG (*n* = 66, 43.7%). After propensity score matching to adjust for baseline clinical characteristics, 96 patients were selected for analysis.

### Baseline and lesion characteristics

The baseline and lesion characteristics of the study patients before and after propensity score matching are summarized in Tables 1 and 2, respectively. The PCI group was significantly older at baseline, while patient characteristics were similar between the two groups after adjustment. Regarding the matched population, lesion complexity and stenosis severity were similar between PCI and CABG groups. Specifically, there were comparable frequencies of LM bifurcation involvement, multivessel disease, and chronic total occlusion, resulting in similar SYNTAX scores between PCI and CABG groups (22.6 ± 10.7 vs. 21.4 ± 7.2, *p* = 0.84). Angiographic stenosis severity was also similar (diameter stenosis: 50.4 ± 15.7% vs. 49.7 ± 8.6%, *p* = 0.36; lesion length: 15.0 ± 7.5 mm vs. 13.5 ± 8.5 mm, *p* = 0.16). Functional stenosis severity alone was significantly greater in PCI group than in CABG group (iFR: 0.79 [0.69–0.85] vs. 0.84 [0.80–0.87], *p* = 0.015).

**Table 1:** Patient and Lesion Characteristics before Propensity Score Matching

	PCI (*n* = 85)	CABG (*n* = 66)	*p* value
Patient characteristics			
Age, yrs	69.8 ± 10.3	63.6 ± 8.9	< 0.001
Male	70 (82.4)	55 (83.3)	0.88
Hypertension	63 (74.1)	48 (72.7)	0.86
Dyslipidemia	65 (76.5)	42 (63.6)	0.11
Diabetes mellitus	43 (50.6)	25 (37.9)	0.14
Chronic kidney disease	25 (29.4)	13 (19.7)	0.19
Current smoker	19 (22.4)	25 (37.9)	0.05
Family history of CAD	9 (10.6)	10 (15.2)	0.46
Previous myocardial infarction	24 (28.2)	19 (28.8)	1.0
Lesion characteristics			
Left main lesion type			
Ostial type	27 (31.8)	16 (24.2)	0.31
Mid type	25 (29.4)	14 (21.2)	0.25
Distal type	72 (84.7)	56 (84.8)	0.98
Other diseased vessels			
No. of diseased vessels			0.51
0	5 (5.9)	8 (12.1)	
1	21 (24.7)	17 (25.8)	
2	38 (44.7)	24 (36.4)	
3	21 (24.7)	17 (25.8)	
LAD	73 (85.9)	48 (72.7)	0.05
LCx	48 (56.5)	29 (43.9)	0.13
RCA	39 (45.9)	39 (59.1)	0.11
With CTO	8 (9.4)	11 (16.7)	0.20
SYNTAX Score	22.6 ± 9.4	22.5 ± 7.1	0.91
Quantitative coronary angiography			
Diameter stenosis, %	49.5 ± 15.7	48.9 ± 10.1	0.79
Minimum lumen diameter, mm	1.80 ± 0.65	1.94 ± 0.60	0.19
Reference diameter, mm	3.61 ± 0.67	3.78 ± 0.76	0.14
Lesion length, mm	14.4 ± 7.8	13.6 ± 8.5	0.59
Physiological stenosis severity			
iFR	0.78 (0.67–0.85)	0.84 (0.76–0.87)	0.014

Values are mean ± standard deviation, *n* (%), or median (interquartile range)

CABG coronary artery bypass grafting, CAD coronary artery disease, CTO chronic total occlusion, iFR instantaneous wave-free ratio, LAD left anterior descending artery, LCx left circumflex artery, PCI percutaneous coronary intervention, RCA right coronary artery

**Table 2:** Patient and Lesion Characteristics after Propensity Score Matching

	PCI (*n* = 48)	CABG (*n* = 48)	*p* value	SMD
Patient characteristics				
Age, yrs	66.5 ± 10.4	66.6 ± 7.9	0.84	0.007
Male	37 (77.1)	39 (82.8)	0.80	0.10
Hypertension	36 (75.0)	35 (72.9)	1.0	0.047
Dyslipidemia	31 (64.6)	31 (64.6)	1.0	< 0.001
Diabetes mellitus	20 (41.7)	18 (37.5)	0.84	0.085
Chronic kidney disease	13 (27.1)	9 (18.8)	0.47	0.19
Current smoker	14 (29.1)	14 (29.2)	1.0	< 0.001
Family history of CAD	5 (10.4)	5 (10.4)	1.0	< 0.001
Previous myocardial infarction	12 (25.0)	9 (18.8)	0.62	0.15
Lesion characteristics				
Left main lesion type				
Ostial type	15 (31.3)	12 (25.0)	0.65	0.14
Mid type	16 (33.3)	6 (12.5)	0.027	0.51
Distal type	38 (79.2)	40 (83.3)	0.79	0.11
Other diseased vessels				
No. of diseased vessels			0.32	0.23
0	5 (10.4)	8 (16.7)		
1	9 (18.8)	11 (22.9)		
2	21 (43.8)	18 (37.5)		
3	13 (27.1)	11 (22.9)		
LAD	40 (83.3)	33 (68.8)	0.15	0.35
LCx	29 (60.4)	20 (41.7)	0.10	0.38
RCA	21 (43.8)	27 (56.3)	0.31	0.25
With CTO	4 (8.3)	7 (14.6)	0.52	0.19
SYNTAX Score	22.6 ± 10.7	21.4 ± 7.2	0.84	0.13
Quantitative coronary angiography				
Diameter stenosis, %	50.4 ± 15.7	49.7 ± 8.6	0.36	0.053
Minimum lumen diameter, mm	1.81 ± 0.63	1.95 ± 0.52	0.13	0.24
Reference diameter, mm	3.70 ± 0.67	3.90 ± 0.80	0.37	0.27
Lesion length, mm	15.0 ± 7.5	13.5 ± 8.5	0.16	0.18
Physiological stenosis severity				
iFR	0.79 (0.69–0.85)	0.84 (0.80–0.87)	0.015	0.56

Values are mean ± standard deviation, *n* (%), or median (interquartile range)

SMD: standardized mean difference. Other abbreviations as in Table 1

### Primary and secondary endpoints

**Fig. 2:**
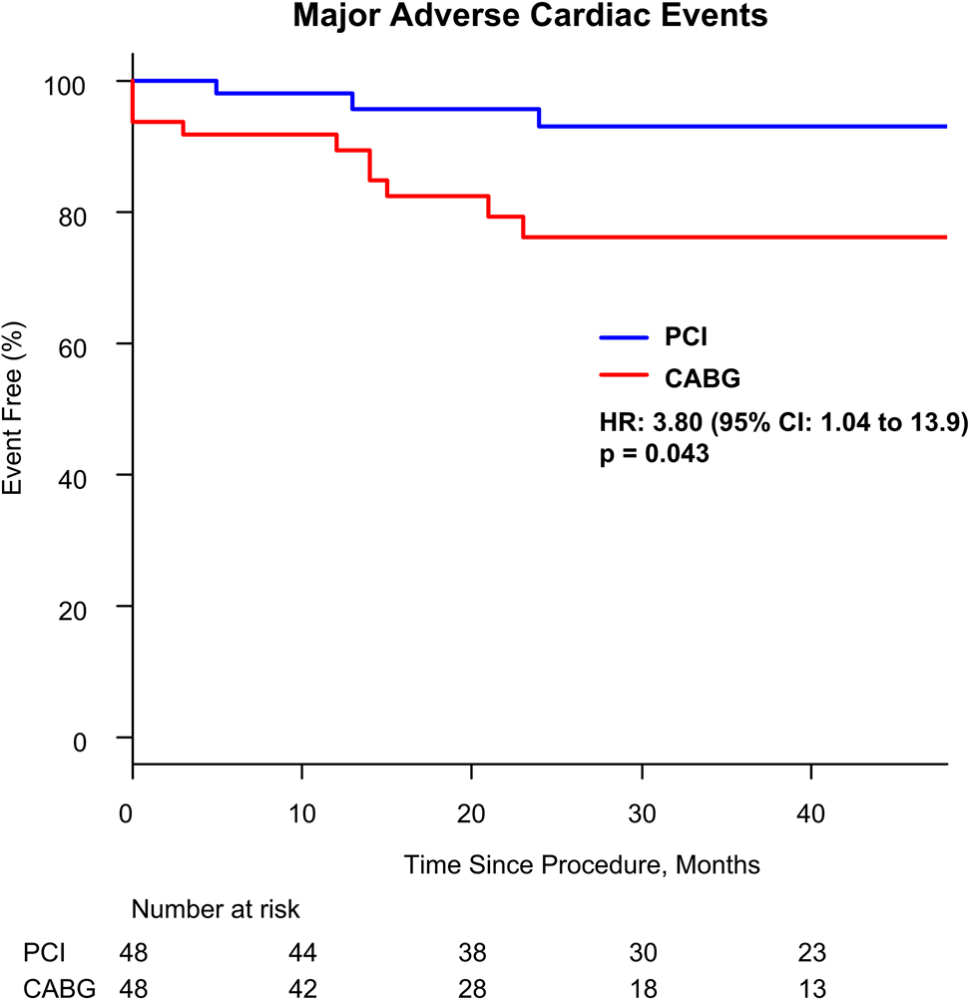
Major adverse cardiac events between iFR-guided PCI vs. CABG. Kaplan–Meier event-free curves showing MACE in the two groups. PCI was significantly associated with lower MACE than CABG. MACE major adverse cardiovascular events. Other abbreviation as in Fig. 1

**Fig. 3:**
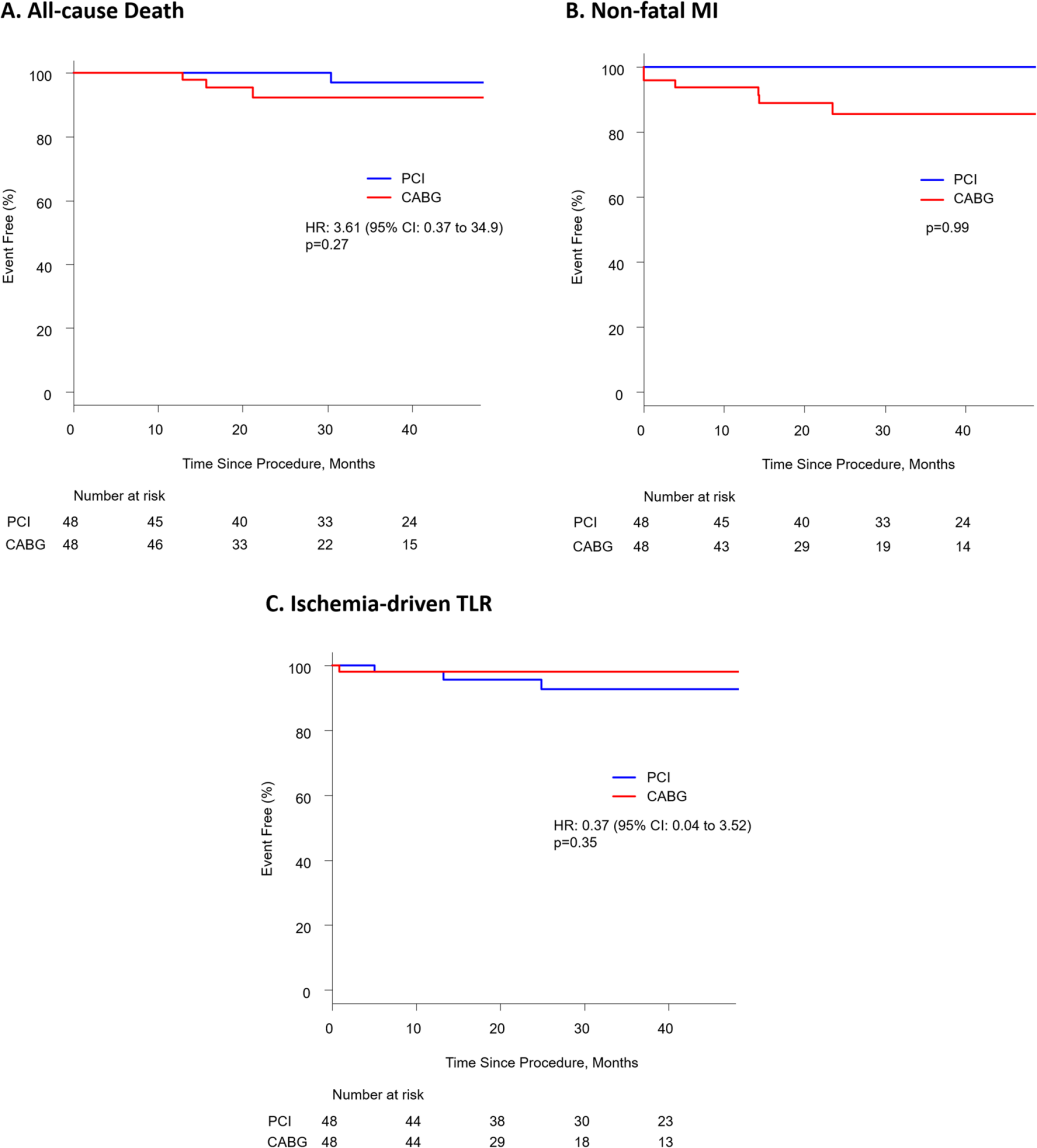
Secondary endpoints between iFR-guided PCI vs. CABG. Kaplan–Meier event-free curves showing **A** all-cause death, **B** non-fatal MI, and **C** ischemia-driven TLR. There were no differences between the two groups. Abbreviation as in Fig. 1

Clinical events for non-adjusted population in this study is demonstrated in Table 3. There were no differences in the rates of MACE between PCI and CABG during follow-up (11/85 [12.9%] vs. 11/66 [16.7%]; HR 1.23; CI 0.53–2.86; *p* = 0.63) despite higher age and lower iFR value in the PCI group with similar SYNTAX score and other patient and lesion characteristics (Table 1). Regarding the matched population, the median follow-up period was 34.3 months (IQR 21.0–46.0). For the primary endpoint, MACE occurred in 4 patients (8.3%) in the PCI group and 10 patients (20.8%) in the CABG group. Kaplan–Meier event-free survival estimates at 4 years demonstrated significantly higher rates of MACE in CABG group (HR 3.80, 95% CI 1.04–13.9, *p* = 0.043) (Fig. 2). For the secondary endpoints, findings in PCI versus CABG groups were as follows: all-cause death: 2.1% vs. 6.3% (HR 3.61, 95% CI 0.37–34.9, *p* = 0.27); non-fatal MI: 0.0% vs. 12.5% (*p* = 0.99); and TLR: 6.3% vs. 2.1% (HR 0.35, 95% CI 0.04–3.34, *p* = 0.37), respectively (Fig. 3). The rates of all-cause death and non-fatal MI were numerically higher in CABG group while the rate of TLR was numerically higher in PCI group. However, none of them were significantly different statistically.

**Table 3:** Clinical Events for Non-adjusted Population

	PCI (*n* = 85)	CABG (*n* = 66)
MACE	11 (12.9)	11 (16.7)
All-cause death	4 (4.7)	3 (4.5)
Non-fatal MI	2 (2.4)	6 (9.1)
TLR	6 (7.1)	2 (3.0)

Values are *n* (%)

MACE major adverse cardiovascular events, MI myocardial infarction, TLR target lesion revascularization. Other abbreviations as in Table 1

In the PCI group, one patient died during study follow-up, of which cause was considered to be cardiac (heart failure). Stent thrombosis and any other MI were not observed. There were three TLRs, of which causes were as follows: in-stent restenosis in distal LM trunk (*n* = 2); and restenosis in ostial LCx after LM-LAD stenting with kissing balloon technique (*n* = 1). Among them, one patient died one year after percutaneous TLR for LM in-stent restenosis (above mentioned cardiac death). In the CABG group, three patients died during study follow-up, of which the causes were cardiac death, pneumonia, and chronic obstructive pulmonary disease. There were six non-fatal MIs, two of which were periprocedural MI and four of which were due to the acute occlusion of saphenous vein grafts to either the LCx or the right coronary artery. One TLR for native LMD was observed due to an occluded left internal mammary artery graft to the LAD.

### Predictive factors of MACE

To investigate potential predictors of MACE, we assessed all patient and lesion characteristics as well as revascularization option (i.e. PCI or CABG) between the cases with MACE (*n* = 13) and those without (*n* = 83) in matched population. In univariate analysis, the performing CABG was significantly predictive for MACE (*p* = 0.03) and the presence of chronic kidney disease and longer lesion had some tendency for the predictability (*p* = 0.054 and *p* = 0.086, respectively). Multivariate analysis revealed that the revascularization option of CABG and the presence of chronic kidney disease were significant predictors for MACE (Table 4). The results of univariate and multivariate analysis for overall population are described in Supplemental Tables S2 and S3.

**Table 4:** Multivariate predictors of major adverse cardiac events after adjustment

Factor	Hazard ratio	95% confidence interval	*p* value
Performing CABG	4.09	1.12–14.9	0.033
Presence of CKD	3.26	1.09–9.74	0.034
Shorter Lesion Length	0.91	0.81–1.03	0.13

For continuous variables, hazard ratios were demonstrated as per 1 unit basis. For nominal variables, hazard ratios were demonstrated with the presence of the factors

CKD chronic kidney disease. Other abbreviation as in Table 1

## Discussion

From the DEFINE-LM registry, representing the largest international registry of ULMD interrogated with iFR to date, the efficacy and safety of iFR-guided LM PCI were demonstrated. The main findings of our study are as follows. First, to the best of our knowledge, this is the only study to determine the long-term clinical outcomes of well-matched ULMD patients with intermediate SYNTAX score revascularized by either PCI or CABG, guided exclusively by iFR. Second, in such a population, the rate of MACE (composite of all-cause death, non-fatal MI, and TLR) was significantly lower in PCI versus CABG patients. Third, there was no significant difference in the individual components of MACE between two treatment options. Although the present analysis was performed retrospectively in a relatively small cohort, the findings of our study are hypothesis-generating to improve the quality of PCI for ULMD.

### Physiology-guided LM revascularization: PCI vs. CABG

There is only limited data available regarding the comparison between PCI versus CABG methods of revascularization for physiologically significant ULMD. This is because such patients have largely been excluded from the majority of randomized controlled trials of physiology-guided revascularization [5–9]. Even the largest pooled meta-analysis of the observational studies of Fractional Flow Reserve-guided ULMD revascularization included PCI as therapeutic option only in 6.0% (13/217) of the study population [14].

Before propensity score matching of the present dataset, as a part of the initial report of DEFINE-LM registry, we had demonstrated comparable outcomes of patients who underwent PCI to those who underwent CABG (MACE: 12.9% [11/85] vs. 16.7% [11/66], HR 1.23, 95% CI 0.53–2.86, *p* = 0.63) despite a relatively higher risk patient population in the PCI group [12]. Specifically, patients in PCI group were significantly older (69.8 ± 10.3 vs. 63.6 ± 8.9, *p* < 0.001) with a numerically higher frequency of diabetes mellitus (50.6% vs. 37.9%, *p* = 0.14). These observed differences in patient demographics reflect real-world clinical practice, where often PCI is selected for higher surgical risk patients, despite known clinical advantages of CABG for ULMD with diabetes mellitus over PCI [15]. Accordingly, the present analysis provides novel insight into the efficacy and safety of contemporary physiology-guided PCI for ULMD applicable to real-world practice.

### Outcomes of the state-of-the-art LM PCI

Since the 1st generation DES era, it is well established that there is no significant difference in mortality rates following revascularization by either PCI or CABG for ULMD [16]. Because of refinements in contemporary PCI practice, including the use of latest generation DES for all PCI cases in the current study, our finding of a reduction in mortality for iFR-guided PCI to ULMD compared to CABG may not be surprising. Furthermore, regarding MI and TLR, it is known that physiology-guided PCI reduces those events compared to angiography-guided PCI practice [5]. This may in part be driven by an overall reduction in the number and length of the stents deployed as a result of physiological assessment. In addition, in the present dataset, all the PCI procedures were optimized by intracoronary imaging modalities as well (usage of intravascular ultrasound in 44 cases and optical coherence tomography in 4 cases), of which advantages had been well demonstrated in terms of the risks of periprocedural/spontaneous MI, stent thrombosis, and TLR [17–19].

Therefore, PCI performed in the present study can be considered synonymous with the “state-of-the-art” SYNTAX-II strategy, which has been demonstrated to deliver improved clinical outcomes over the SYNTAX-I PCI cohort and comparable to CABG in three-vessel disease patients [3, 4].

### Potential procedural benefit from iFR-pullback

As described in the result, bifurcation lesions were predominant: 79.2% (38/48) of the cases in PCI group. Among them, complex bifurcation lesions, i.e. Medina classification (1,1,1), (1,1,0), (1,0,1), and (0,1,1) were observed in 73.7% (28/38) and presence of the ostial lesion in the LCx was observed in 44.7% (17/38) of the cases on angiographical findings (Supplemental Table S1). However, two-stent technique was required for only two cases.

**Fig. 4:**
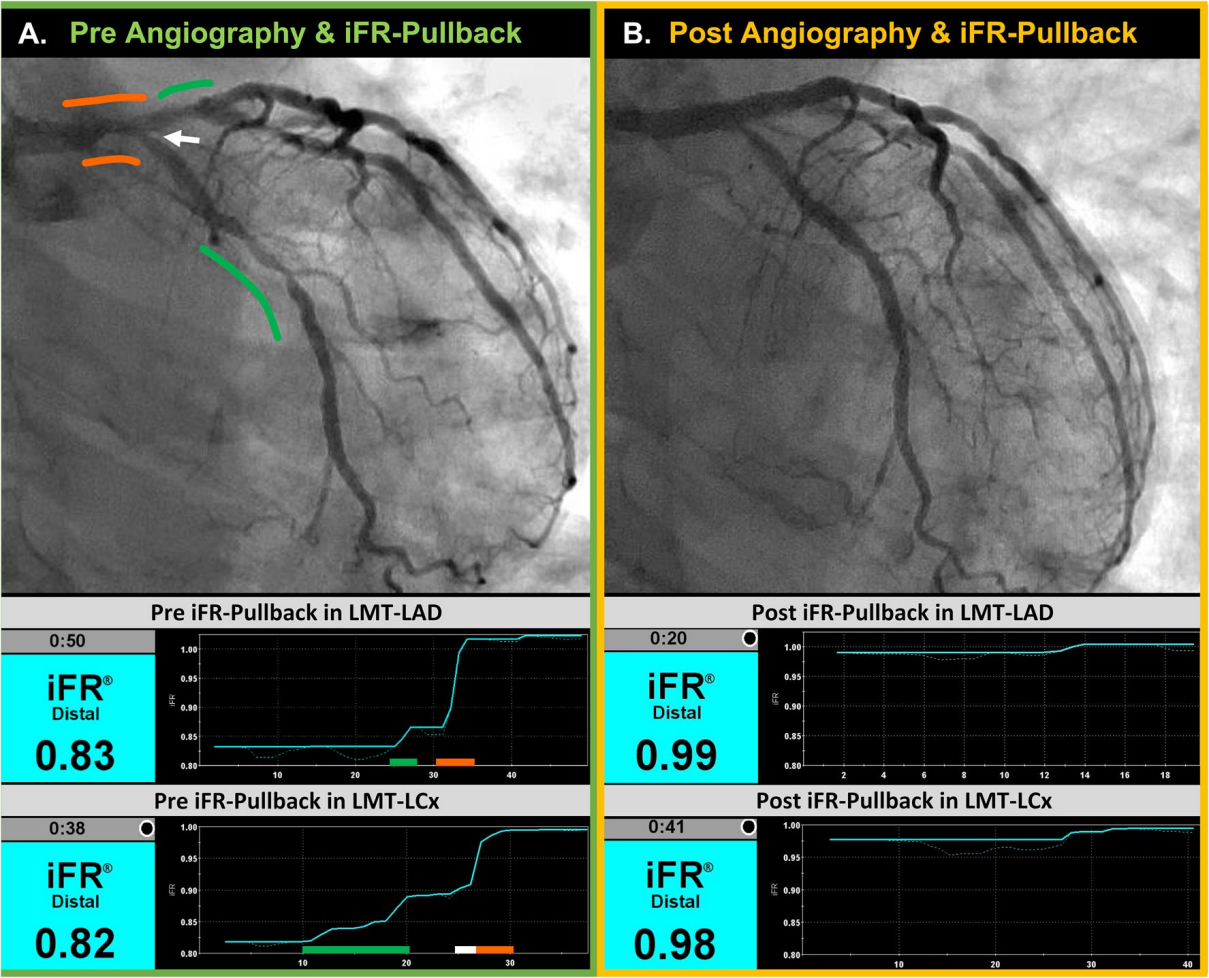
Representative Case of iFR-Pullback Guided LM PCI. **A** Pre-PCI angiography and iFR-pullback curves. In the upper panel, angiography is shown with overlaid images of pressure gradient co-registration obtained by iFR-pullbacks from LAD and LCx. In the lower panel, iFR-pullback curves from LAD and LCx are demonstrated. Green lines denote pressure gradient in the downstream vessels of LMT, co-registered in angiography with green curves. Orange lines denote pressure gradient in LMT, co-registered in angiography with orange curves. A white line denotes small pressure gradient (⊿iFR = 0.01) in the ostial lesion of the LCx, co-registered in angiography with a white arrow. **B** Post-PCI angiography and iFR-pullback curves. In the upper panel, angiography shows well-expanded stent in LMT-LAD and mild stenosis in the ostium of LCx. Far distal iFR values in both LAD and LCx were not significant. Small pressure gradients were confirmed within the LM stent and the LCx ostial lesion, respectively (each ⊿iFR = 0.01). LAD left anterior descending artery, LCx left circumflex artery, LMT left main trunk. Other abbreviation as in Fig. 1

In this regard, we speculate that iFR-pullback guided decision-making would be effectively utilized in some cases to detect the specific lesion to be revascularized [20]. The representative case using iFR-pullback in the present study is shown in Fig. 4. Based on angiographic findings, two-stent technique for LM bifurcation lesion might be performed due to the moderate stenosis in LCx ostium. However, iFR-pullback clearly demonstrated the absence of physiological significance in the lesion (Fig. 4A, a white arrow, ⊿iFR = 0.01 in this segment). Accordingly, single-stenting with kissing balloon technique was performed and consequently, angiographically acceptable and physiologically excellent result was obtained (Fig. 4B). Details of the procedure are described in Supplemental Fig. S1. As a result, MACE-free for 45 months was confirmed in this case.

The ostial lesion of the LCx is known to be unfavorable entity for PCI with higher risk of TLR, especially in two-stent technique [21]. Conversion to the two-stent technique from provisional stenting further diminishes the value of LM PCI in complex bifurcation lesion [22]. Thus, avoiding unnecessary intervention for the LCx ostial lesion would be preferable if possible. Although the magnitude of impact of iFR-pullback guidance was unclear in this study due to the study design, such a strategy would have contributed more simple procedure and following favorable outcomes considerably. Accordingly, we expect the iFR-pullback based strategy to be a gatekeeper to appropriately avoid Achilles’ heel of PCI (LCx ostial lesion). Further studies are warranted to confirm this speculation regarding the efficacy of iFR-pullback in LM PCI raised by the present study.

### Impact of coronary physiology on graft patency

Although the available data is limited, several observational studies have reported that the patency of bypass grafts for physiologically non-significant stenoses as being inferior than for physiologically significant ones [23–25]. A recent report demonstrated that higher preoperative iFR was significantly associated with an increased risk of graft failure within 1 year after CABG (HR per iFR unit increase: 1.11; 95% CI 1.03–1.19; *p* = 0.003) [25]. In that study, the cutoff value of iFR to predict graft failure was determined as 0.84 by receiver-operating characteristic analysis. Of note, in our study, median iFR value in the CABG group was also 0.84 (IQR 0.80–0.87). This may in part account for the MACE observed in this group with one TLR occurring due to the occlusion of the left internal mammary artery graft to LAD (1/1 TLR); and three non-fatal MIs due to the acute thrombotic occlusion of saphenous vein graft to LCx (3/6 non-fatal MI). Considering such impact of preoperative iFR on bypass graft failure, it might be understandable that performing CABG was a predictive factor for MACE in this dataset.

### Study limitations

The present study has several limitations. First, the study size was relatively small, which might have affected the statistical significance or non-significance. The difference in distributions of propensity score between PCI and CABG groups before adjustment might further reduce the number of matched cases (Supplemental Fig. S2). Further studies should validate the current results and warrant the efficacy and safety of iFR-guided LM PCI in randomized controlled designs or larger registry studies.

Second, due to the non-randomized nature of this study, a potential for selection bias of iFR measurement for ULMD must be considered. More complex cases, such as ULMD with severe stenosis in LCx ostium, which is not favorable for PCI as we discussed, might have been treated by CABG without iFR interrogation and thus not been included in this registry. However, the frequency of complex bifurcation ULMD in PCI group was not low in this study (Supplemental Table S1). Furthermore, the strength of a registry-based approach is reflecting that it reflects the patient population in real-world clinical practice (e.g. an inexorable choice of PCI despite comorbidity of diabetes mellitus due to old age, as shown in Table 1 and Supplemental Fig. S3).

Third, the nature of the present analysis leaves room for residual confounding despite performing propensity score matching. There were unmeasured confounding factors: perioperative risk factors, cardiac function, comorbidity of heart failure, chronic pulmonary disease, and liver and other organ dysfunction.

Forth, the present results could not be extrapolated to the patients with high SYNTAX score ≥ 33. CABG is strongly recommended for those patients in international guidelines [1, 2].

Fifth, 100% usage of intracoronary imaging devices in PCI group occurred coincidently. Despite this, because the protocol did not mandate its usage, the degree of imaging-guided stent optimization and the magnitude of impact on PCI outcomes cannot be evaluated.

Sixth, the details of the CABG procedure are unknown though the internal thoracic artery grafts are routinely used for LAD in all the participated centers.

Seventh, we could not provide details of medical therapy and risk factor control over the follow-up period in both groups. However, as per routine clinical practice, guideline-directed medical therapy was applied as normal in each participating center.

Finally, other several limitations should be acknowledged. Clinical events were recorded and reported by each participating center without an independent clinical events committee to adjudicate events. We could not provide details of medical therapy and risk factor control over the follow-up period. Furthermore, quantitative coronary angiography analysis was not performed at an independent core laboratory.

## Conclusions

Within this propensity score matched sub-analysis of the DEFINE-LM registry, representing the largest international registry of ULMD cases interrogated with iFR, iFR-guided PCI was associated with a lower risk of long-term clinical outcomes compared with CABG in patients with intermediate SYNTAX score and physiologically significant ULMD. Although this study has several limitations to be conclusive, the present results are hypothesis-generating to improve the quality of LM PCI. Further studies should warrant the value of physiology-guided LM PCI in randomized controlled designs or larger registry studies.

## Supplementary Information

Below is the link to the electronic supplementary material.

Supplementary file1 (DOCX 1359 KB)

## Data Availability

The authors declare that all supporting data are available within the article and its online supplementary files.
