# Supplementary material for: Physiology-guided PCI versus CABG for left main coronary artery disease: insights from the DEFINE-LM registry
Source: Cardiovasc Interv Ther. 2023 Apr 5;38(3):287–98. doi: 10.1007/s12928-023-00932-z (PMC10247826; doi:10.1007/s12928-023-00932-z)
Supplement: Supplementary file 1 — Supplementary file1 (DOCX 1359 KB) [file 12928_2023_932_MOESM1_ESM.docx]

**- Supplementary File -**

**Supplemental Tables.**

**Table S1. Distribution of Bifurcation Lesion According to Medina Classification.**

| **Medina classification** | **PCI (n=38), n (%)** | **CABG (n=40), n (%)** |
| --- | --- | --- |
| (1,1,1) | 10 (26.3) | 13 (32.5) |
| (1,1,0) | 11 (28.9) | 11 (27.5) |
| (1,0,1) | 5 (13.2) | 1 (2.5) |
| (1,0,0) | 5 (13.2) | 7 (17.5) |
| (0,1,1) | 2 (5.3) | 4 (10) |
| (0,1,0) | 5 (13.2) | 4 (10) |
| (0,0,1) | 0 (0) | 0 (0) |

Distal left main bifurcation lesions were observed in 79.2% (38/48) of the patients among PCI group and in 83.3% (40/48) of the patients among CABG group, respectively.

CABG: coronary artery bypass grafting; PCI: percutaneous coronary intervention.

**Table S2.** **Univariate Predictors of Major Adverse Cardiac Events before Adjustment.**

| **Factor** | **Hazard Ratio** | **95% Confidence Interval** | **p value** |
| --- | --- | --- | --- |
| Age | 1.06 | 0.99-1.13 | 0.072 |
| Male | 0.54 | 0.16-1.84 | 0.33 |
| Hypertension | 1.69 | 0.34-8.46 | 0.53 |
| Dyslipidemia | 1.46 | 0.39-5.53 | 0.58 |
| Diabetes mellitus | 2.64 | 0.75-9.39 | 0.13 |
| Chronic kidney disease | 4.31 | 1.56-11.9 | 0.005 |
| Current smoker | 4.44 | 1.40-14.1 | 0.011 |
| Family history of CAD | 0.54 | 0.09-3.32 | 0.51 |
| Previous MI | 1.05 | 0.35-3.14 | 0.93 |
| Distal LM disease | 16.9 | 1.22-234.8 | 0.035 |
| No. of diseased vessel | 1.18 | 0.86-1.63 | 0.31 |
| Isolated LM disease | 1.31 | 0.14-12.5 | 0.81 |
| LAD | 0.27 | 0.05-1.43 | 0.12 |
| LCx | 1.57 | 0.52-4.70 | 0.42 |
| RCA | 0.85 | 0.22-3.26 | 0.81 |
| With CTO | 0.68 | 0.18-2.67 | 0.58 |
| SYNTAX Score | 1.02 | 0.95-1.09 | 0.57 |
| % Diameter stenosis | 0.90 | 0.78-1.04 | 0.15 |
| Lesion length | 0.88 | 0.79-0.97 | 0.011 |
| iFR | 22.04 | 0.18-2718.0 | 0.21 |
| Performing CABG | 2.63 | 0.66-10.5 | 0.17 |

CAD: coronary artery disease; CTO: chronic total occlusion; iFR: instantaneous wave-free ratio; LAD: left anterior descending artery; LCx: left circumflex artery; LM: left main; RCA: right coronary artery. Other abbreviations as in Table S1.

**Table S3.** **Multivariate Predictors of Major Adverse Cardiac Events before Adjustment.**

| **Factor** | **Hazard Ratio** | **95% Confidence Interval** | **p value** |
| --- | --- | --- | --- |
| Age | 1.05 | 0.99-1.09 | 0.067 |
| Presence of CKD | 3.10 | 1.27-7.53 | 0.012 |
| Current Smoker | 2.21 | 0.90-5.41 | 0.08 |
| Shorter Lesion Length | 0.92 | 0.85-0.99 | 0.022 |

For continuous variables, hazard ratios were demonstrated as per 1 unit basis. For nominal variables, hazard ratios were demonstrated with the presence of the factors.

CKD: chronic kidney disease.

**Supplemental Figures.**

**Figure S1. Procedure of iFR-pullback Guided Left Main Bifurcation PCI.**


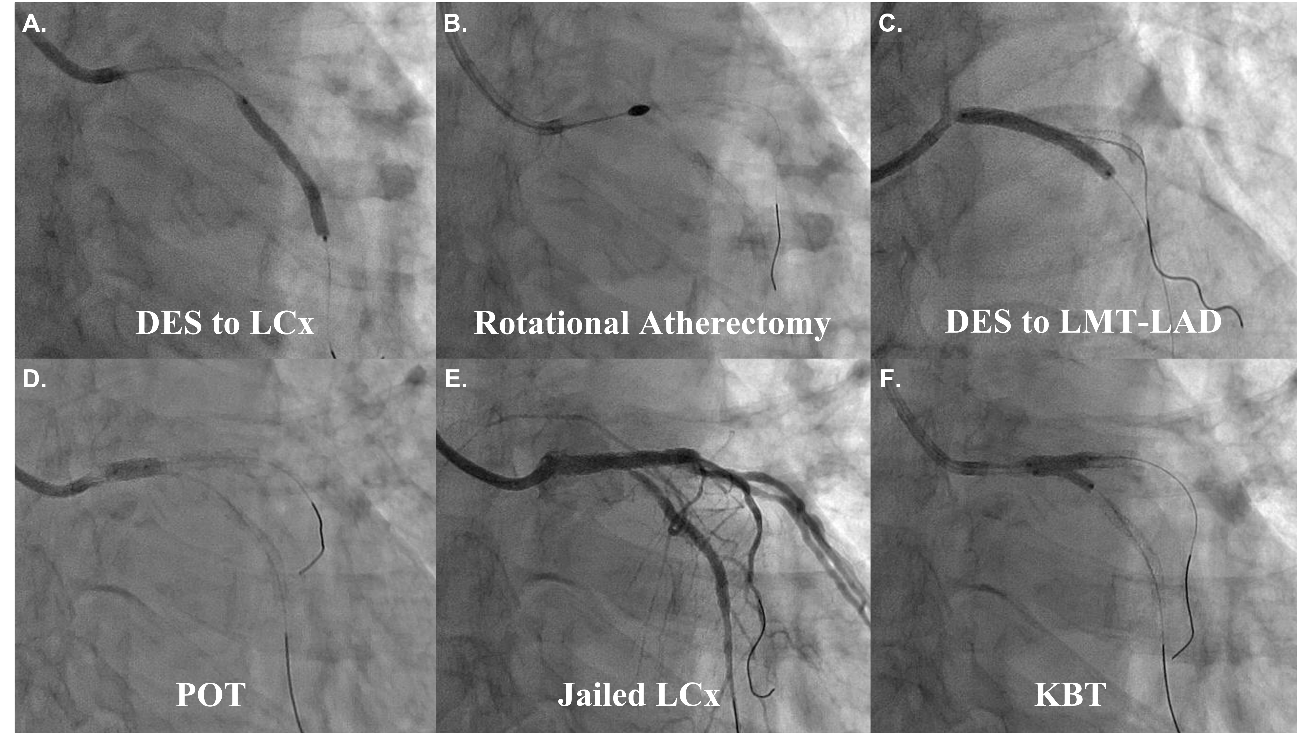


A. Based on pressure gradient in LCx, a DES was deployed followed by post-dilatation.

B. Due to heavily calcified lesion in LMT-LAD, rotational atherectomy was performed.

C. Crossover stenting across LMT-LAD was performed with wire-protection to LCx.

D. POT with a large non-compliant balloon was performed.

E. Severe stenosis was confirmed at the ostium of LCx.

F. KBT was performed following wire recross to LCx.

The procedure was guided by intravascular ultrasound as well.

DES: drug-eluting stent; iFR: instantaneous wave-free ratio; KBT: kissing balloon technique; LAD; left anterior descending artery: LCx: left circumflex artery; LMT: left main trunk; PCI: percutaneous coronary intervention; POT: proximal optimization.

**
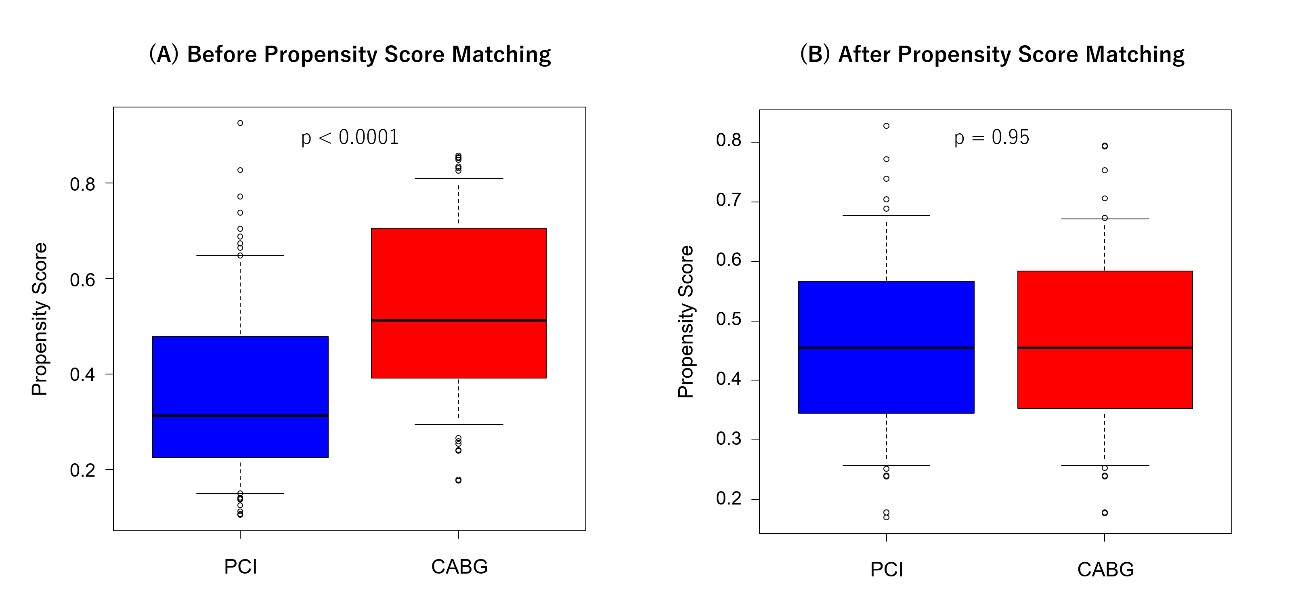
Figure S2. Distribution of Propensity Score.**

Distribution of propensity score before (A) and after (B) adjustment.

CABG: coronary artery bypass grafting; PCI: percutaneous coronary intervention.

**Figure S3. Distributions of Age between PCI and CABG Groups.**


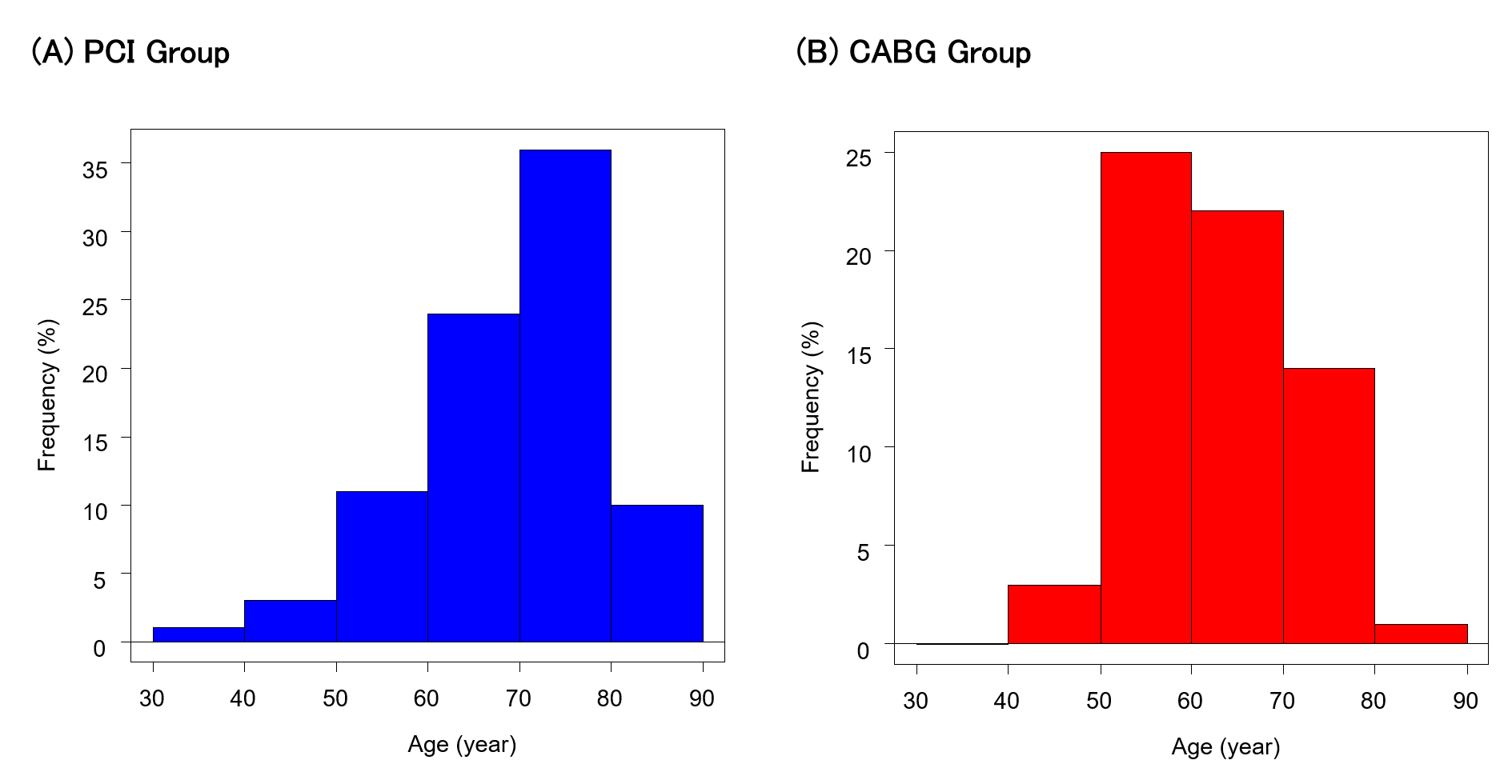


Frequency histograms of age in (A) PCI and (B) CABG groups.

Abbreviations as in Figure S2.
